# Conservative Endodontic Management of Type II Dens Invaginatus With Talon Cusp in a Maxillary Central Incisor: A 2‐Year Follow‐Up Case Report

**DOI:** 10.1002/ccr3.72306

**Published:** 2026-03-13

**Authors:** Sajedeh Namaei Ghasemi, Paria Molaei

**Affiliations:** ^1^ Assistant Professor of Endodontics, Dental Research Center, Faculty of Dentistry Mashhad University of Medical Sciences Mashhad Iran; ^2^ Post‐Graduate Student of Endodontics, Department of Endodontics, Faculty of Dentistry Mashhad University of Medical Sciences Mashhad Iran

**Keywords:** CBCT, dens invaginatus, dental microscope, talon cusp

## Abstract

Dens invaginatus (DI) is a developmental dental anomaly that complicates diagnosis and treatment due to its varied morphology and potential pulpal and periapical involvement. This case report presents the successful endodontic management of a Type II DI in a 14‐year‐old female patient, affecting the maxillary left central incisor, which also exhibited a talon cusp. The patient was asymptomatic, and the lesion was discovered during orthodontic evaluation. Cone‐beam computed tomography (CBCT) revealed an enamel‐lined invagination extending into the root canal system without communication with the periodontal ligament, confirming a Type II DI. Clinical tests indicated pulp necrosis. A conservative treatment plan was adopted, targeting only the main canal and leaving the invaginated portion untreated. Modern diagnostic tools, including CBCT imaging and a dental operating microscope, facilitated accurate diagnosis and precise execution of the treatment. Follow‐up at 3 months and two years showed complete healing of the periapical lesion, and the patient remained asymptomatic. This case highlights the efficacy of a minimally invasive, selective treatment strategy supported by advanced imaging and magnification in managing DI.

**Key Clinical Message**

Conservative endodontic management of Type II dens invaginatus, combined with modern diagnostic tools like CBCT and dental operating microscopes, can effectively preserve tooth structure and resolve periapical pathology, offering a minimally invasive, long‐term solution with favorable clinical outcomes.

## 1 Introduction

Dens invaginatus (DI) is a developmental anomaly that occurs when the enamel organ folds into the dental papilla during early odontogenesis [1]. As mineralization progresses, this can result in an enamel‐lined pocket or cavity that extends variably into the root structure. Developmental anomalies such as DI can affect the shape, size, color, number, and overall formation of teeth, irrespective of whether they are part of the deciduous or permanent dentition. Its formation is associated with the infolding of Hertwig's epithelial root sheath, with development sometimes extending into the root after initial tooth formation is complete [2, 3]. DI can affect primary, permanent, or supernumerary teeth, with a prevalence ranging from 0.04% to 10%. Males are more frequently affected than females, with a male‐to‐female ratio of 3:1 [1]. The condition can occur in any tooth in the maxillary or mandibular arch, but maxillary lateral incisors are the most commonly involved. Other commonly affected teeth include permanent central incisors, premolars, canines, and molars [1].

Teeth with DI often exhibit deep invaginations that are difficult to clean and can harbor plaque and bacteria, leading to caries, pulp necrosis, or periapical lesions without overt clinical symptoms. If left untreated, these anatomical irregularities can compromise the tooth's long‐term prognosis and complicate both preventive and endodontic treatment approaches. The bulbous appearance of the crown may also compromise aesthetics, often prompting patients to request corrective treatment [4].

There are multiple ways to classify DI, depending on the extent of the invagination. Oehler's system is the most widely recognized. Type I is characterized by a minor enamel‐lined invagination confined to the crown. In Type II, the invagination remains enamel‐lined but extends into the pulp chamber without establishing communication with the periodontal ligament. Type III involves the invagination extending through the root. Type IIIA has lateral communication with the periodontal ligament via a pseudoforamen, while Type IIIB features an invagination that extends through the root to the apical foramen, establishing communication with the periodontal ligament but without any connection to the pulp [5, 6].

This case describes the diagnosis and conservative management of a maxillary central incisor with Type II DI and a talon cusp. The treatment focused on preserving uninvolved structures while resolving periapical pathology.

## 2 Case History/Examination

**FIGURE 1:**
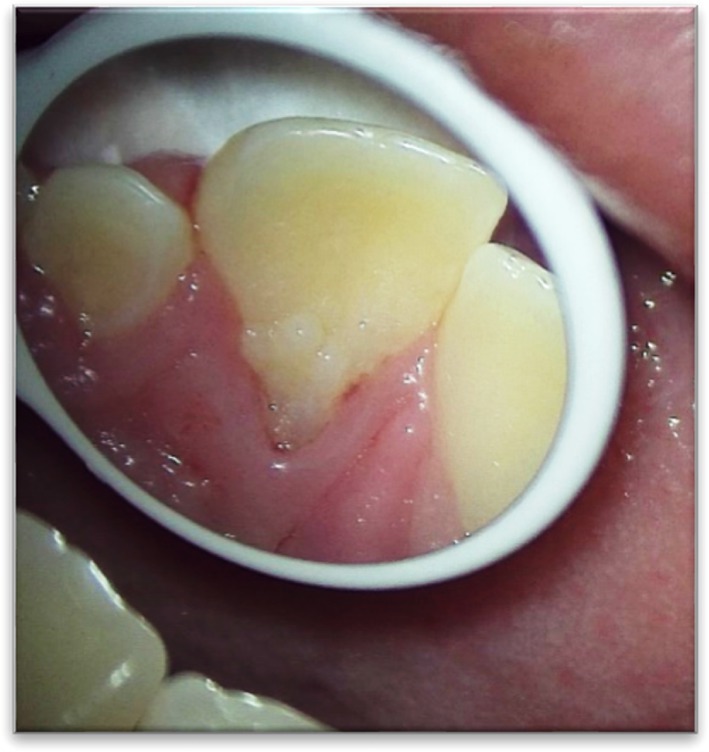
Initial clinical view.

A 14‐year‐old female was referred to the Department of Endodontics at Mashhad University of Medical Sciences (MUMS) for evaluation of an incidental radiolucency discovered during orthodontic treatment planning. The lesion was associated with the maxillary left central incisor (tooth #9). (Figure 1) The patient was medically healthy.

Clinical examination showed a talon cusp on tooth #9 and a peg‐shaped lateral incisor (tooth #10). (Figure 1) Pulp vitality tests, including cold, heat, and electric pulp testing (EPT), were negative for tooth #9, which was slightly tender to percussion but not to palpation. Periodontal probing around tooth #9 revealed a 3 mm pocket depth, with no bleeding on probing.

**FIGURE 2:**
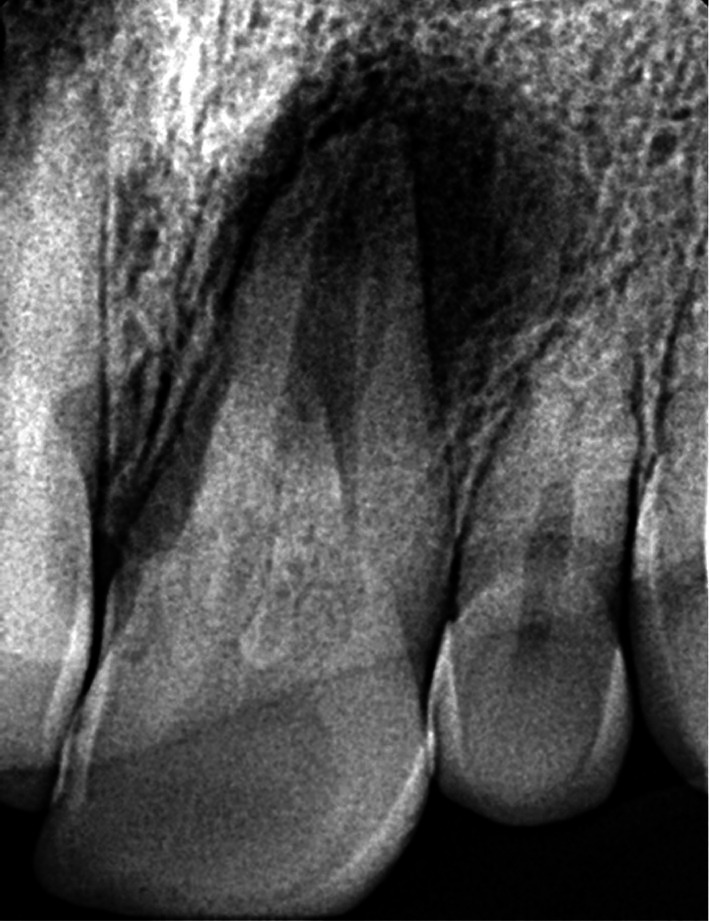
Initial radiographic view.

**FIGURE 3:**
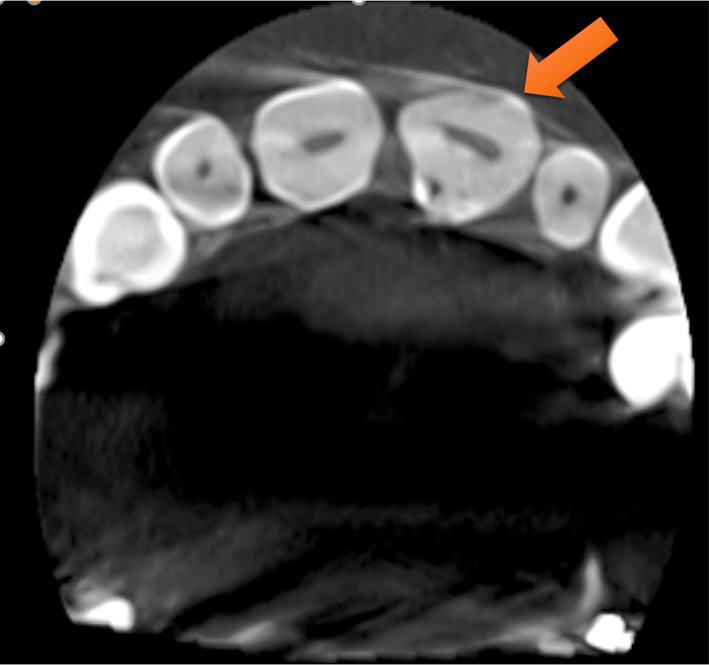
Axial view of CBCT.

**FIGURE 4:**
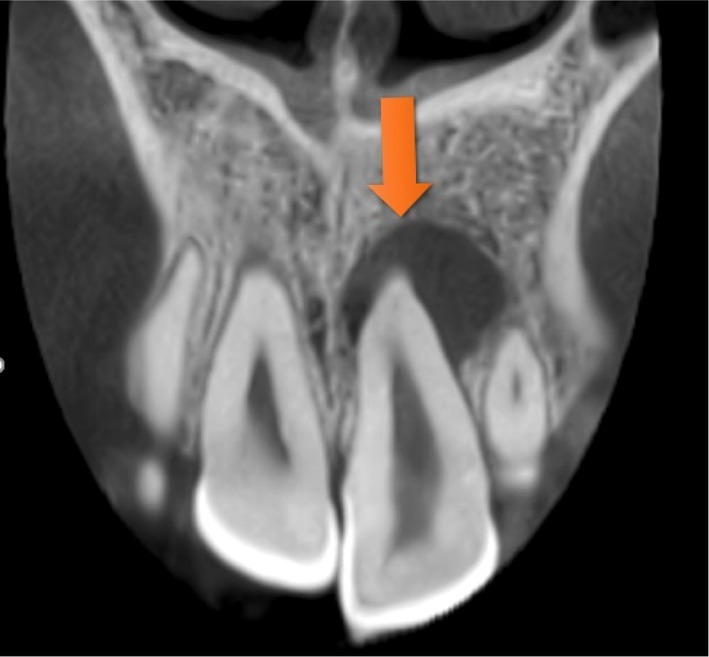
Coronal view of CBCT.

**FIGURE 5:**
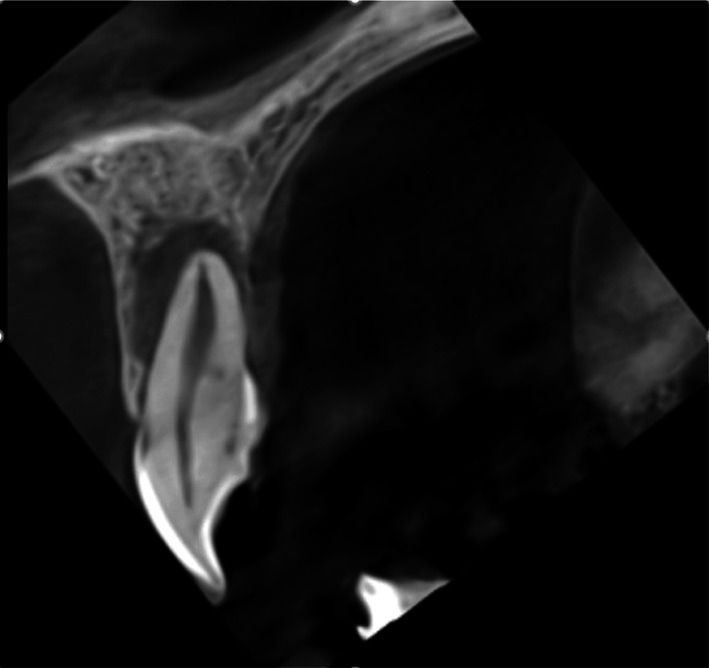
Sagittal view of CBCT.

Radiographic analysis revealed a periapical radiolucency at the apex of tooth #9, along with widening of the periodontal ligament (PDL) space. (Figure 2) CBCT was also ordered, which confirmed the presence of Oehlers Type II dens invaginatus, confined to the crown of the tooth, with no radiolucency observed around the invaginated portion, indicating that the invagination was not involved in infection or inflammation (Figures 3, 4, 5).

Given these findings, the patient was diagnosed with pulp necrosis and associated symptomatic apical periodontitis in tooth #9.

## 3 Case Description

The treatment plan involved non‐surgical root canal therapy for tooth #9, addressing the periapical pathology while leaving the invaginated portion of the tooth untreated, as it was clinically unaffected. The treatment plan was explained to the patient, and informed consent was taken.

**FIGURE 6:**
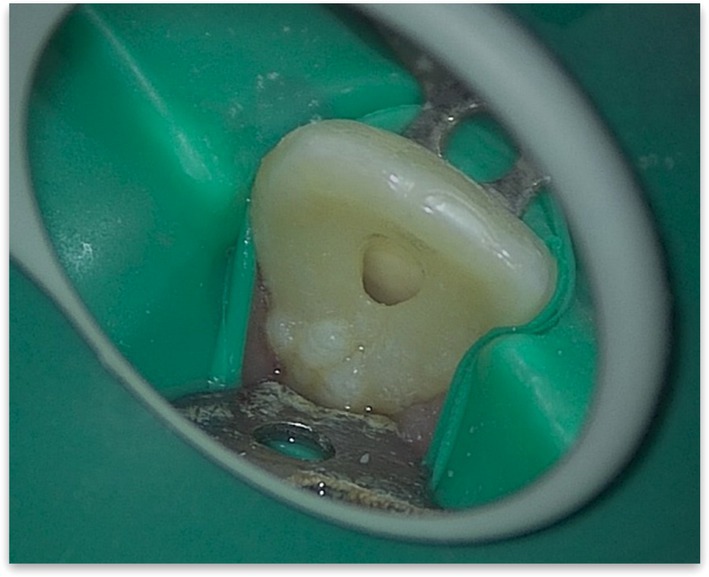
Access cavity.

**FIGURE 7:**
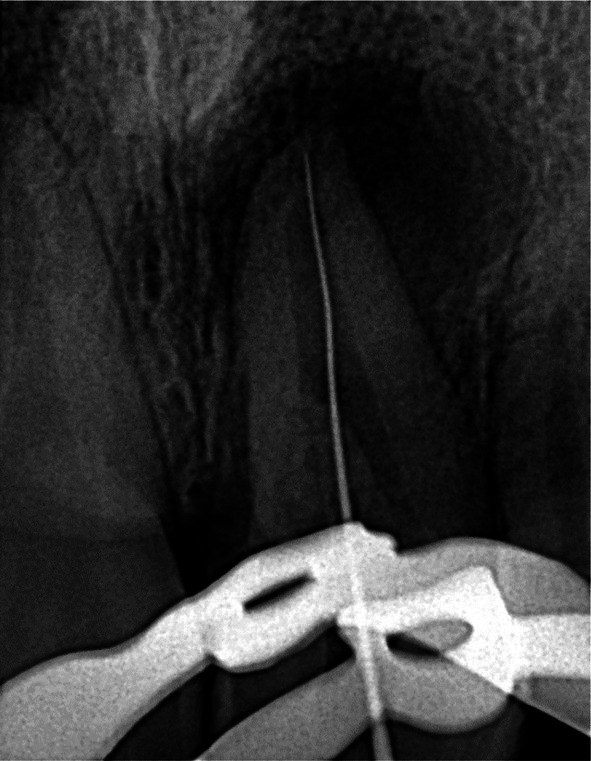
Initial file radiography.

In the first treatment session, the patient was anesthetized using infiltrative local anesthesia (1.8 mL of lidocaine 2% with epinephrine). Rubber dam isolation was applied. An access cavity was prepared using a small round diamond bur and a safe‐ended diamond bur under magnification with a dental operating microscope (Zumax, China) (Figure 6). The working length was determined using the Propex IQ apex locator (Dentsply Sirona, USA) and was confirmed radiographically, with a working length of 24.5 mm. (Figure 7) The canal was patent with a #10 K file (Mani, Japan). The canal was shaped up to 25/.04 (M3, China) with the standard technique. The canal was irrigated copiously with 5.25% sodium hypochlorite (NaOCl) and saline. Calcium hydroxide paste (Prevest DenPro, China) was placed as an intracanal medicament, and the patient was instructed to return in one week.

**FIGURE 8:**
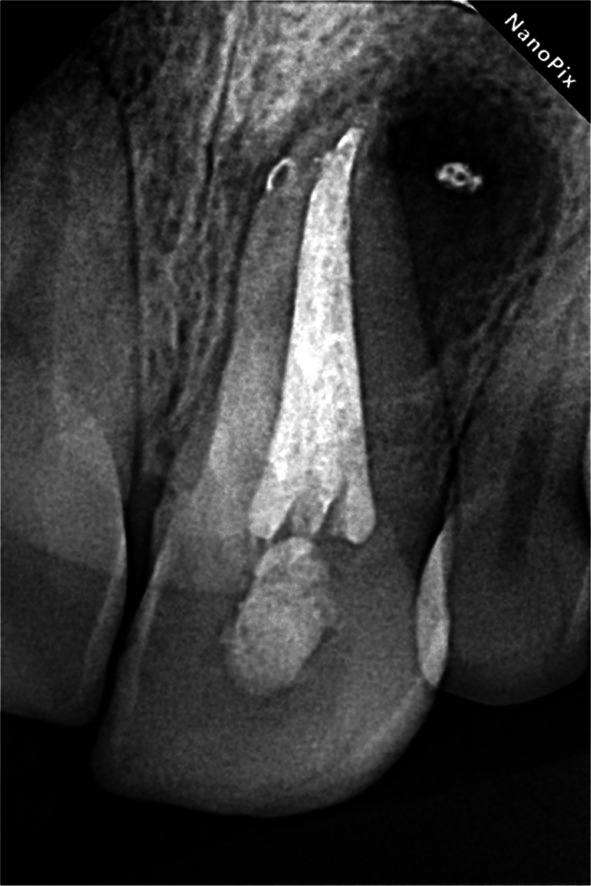
Final radiography.

During the second visit, the canal was irrigated with NaOCl, followed by 17% EDTA for one minute to remove the smear layer. The canal was then dried with sterilized paper points (Meta Biomed, Korea). A hybrid obturation technique was employed, combining cold lateral condensation with 2% gutta‐percha (Meta Biomed, Korea) and warm vertical condensation using Fast‐Pack and Fast‐Fill (Eighteenth, China). AH+ sealer (Dentsply Sirona, USA) was used for the obturation. (Figure 8) The access cavity was temporarily sealed by Coltosol (Asia Chemi Teb, Iran).

Following the procedure, the patient was referred to the restorative dentist for definitive restoration of tooth #9 and to the orthodontist to continue with her orthodontic treatment.

## 4 Conclusions and Results

**FIGURE 9:**
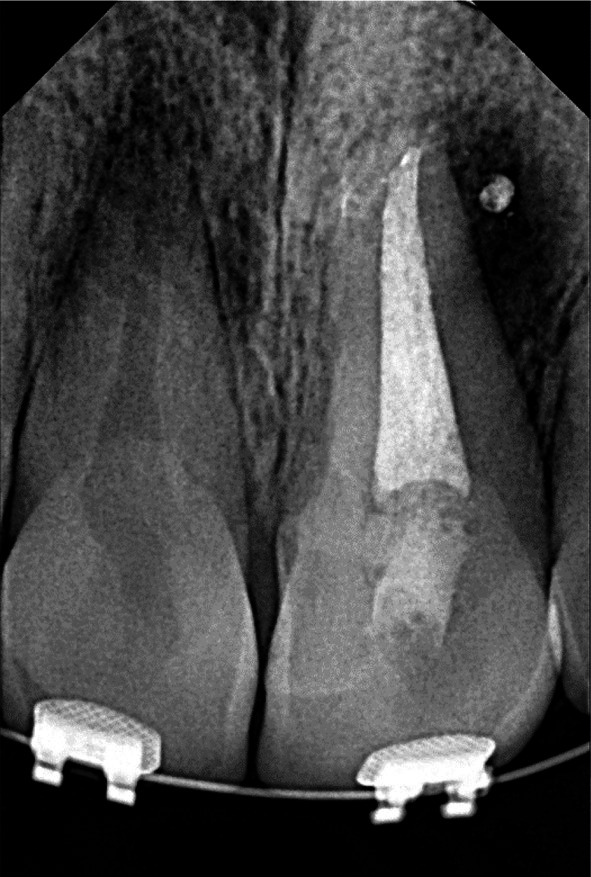
3‐month follow‐up.

**FIGURE 10:**
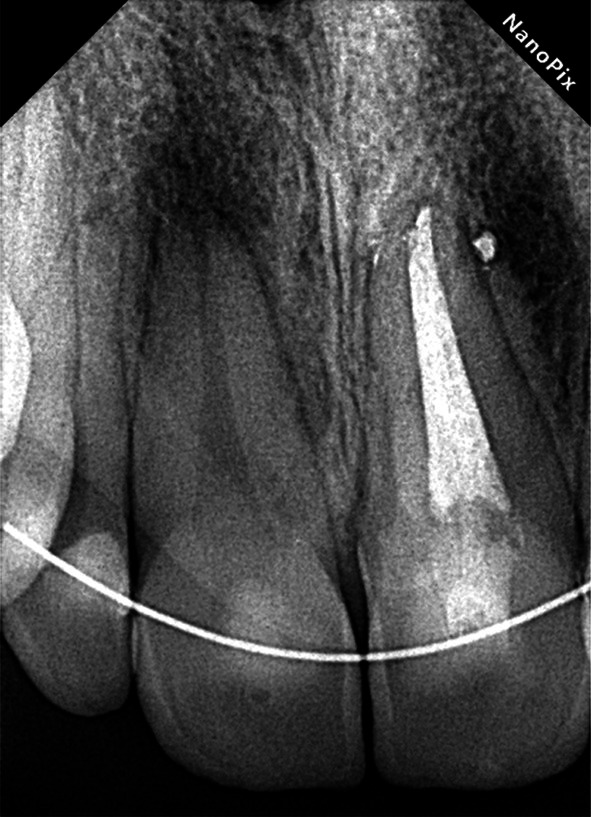
24‐month follow‐up.

The three‐month follow‐up showed the patient asymptomatic with signs of periapical healing, and the two‐year follow‐up confirmed complete healing with no symptoms, demonstrating the long‐term success of the conservative treatment. (Figures 9 and 10) This case highlights the effective management of Type II Dens Invaginatus using a minimally invasive approach, combining CBCT imaging and a dental microscope for non‐surgical root canal therapy. The favorable outcomes after 3 and 24 months of follow‐up emphasize the effectiveness of selective root canal treatment in preserving tooth integrity and achieving lasting results.

## 5 Discussion

Dens invaginatus (DI) presents diagnostic challenges due to its varied morphology and potential impact on the pulp and periapical tissues. Though most commonly found in lateral incisors, this case involved a central incisor with an unusual combination of DI and a talon cusp. Conservative treatment was justified by the absence of pathology in the invaginated portion, which allowed for preservation of tooth structure and avoidance of unnecessary intervention.

Various treatment modalities for DI have been reported in the literature. Some studies treated only the invaginated portion, leaving the main canal unaffected. Schwartz and Schindler (1996) [7], Tsurumachi (2010) [8], and Dembinskaite et al. (2018) [9], described cases where the invaginated area was treated while the main canal remained vital using lateral condensation with gutta‐percha for obturation, similar to our case.

In contrast, other reports suggested treating both the invaginated portion and the main canal. Zhang and Wei (2022) [10], and Bahmani et al. (2020) [11], used ultrasonic tips to remove the invaginated portion before treating and obturating the main canal. However, Zhang and Wei (2022) [10], observed that, despite initial non‐surgical treatment, periapical surgery was required due to persistent symptoms.

Arora et al. (2022) [6], treated both the invaginated portion and the main canal in a Type IIIB maxillary lateral incisor, using lateral compaction and warm vertical obturation techniques. This approach mirrors our treatment plan, where only the main canal was treated and obturated, while the invaginated portion was left untouched. This non‐invasive strategy proved effective in cases where the invagination was not infected, showing promising results.

Some studies in the literature employed unconventional methods. For example, Sawhney et al. (2023) [12], conducted prophylactic pit and fissure sealing in a case of maxillary lateral incisor without endodontic involvement, emphasizing the importance of recognizing when endodontic treatment is unnecessary and when preventive measures can be adopted.

In severe cases with infection or significant symptoms, surgical intervention was necessary. Clarke et al. (2016) [4], treated a Type III maxillary permanent canine with a multidisciplinary approach, while Dembinskaite et al. (2018) [9], performed surgical treatment on the invaginated portion in a Type III case. This demonstrates the need for surgical removal in certain instances. Additionally, Wei et al. (2020) [13], reported the extraction of a tooth due to severe invagination and associated complications, which may remain the best option when conventional treatments are not effective.

Badran et al. (2021) [14], employed a surgical approach for a maxillary central incisor with an atypical dental invagination, reinforcing the role of surgery when non‐surgical methods fail to achieve results. In a case series by Wei et al. (2020) [13], three treatment plans were explored, including management of both the invagination and the main canal, removal of the invaginated part, and intentional replantation. This highlights the variability in treatment approaches based on the type and severity of the invagination, as well as the patient's symptoms and lesion extent.

In this case, CBCT imaging played a pivotal role in diagnosis and treatment planning. Although the initial periapical radiograph revealed an invagination and a periapical radiolucency, it was insufficient to determine the full extent and type of the anomaly. CBCT was therefore asked to provide a detailed three‐dimensional assessment of the invagination, its relationship with the pulp space, and any possible communication with the periodontal ligament or apical tissues. This information was critical for confirming the diagnosis of Oehlers Type II dens invaginatus and for guiding a conservative treatment approach. Selective root canal treatment—targeting only the necrotic main canal while preserving the uninvolved invaginated portion—was chosen to maintain structural integrity and biological function. The success of this approach was greatly enhanced by the use of a dental operating microscope, which provided optimal illumination and magnification. This enabled precise identification of the canal anatomy, improved visualization during access and instrumentation, and minimized the risk of iatrogenic damage. Together, CBCT and enhanced magnification were essential in facilitating accurate diagnosis, conservative management, and ultimately, a favorable long‐term clinical outcome.

Minimally invasive techniques, such as selective endodontics, offer several key benefits. These methods minimize the risk of iatrogenic damage, reduce post‐treatment discomfort, and enhance the overall healing process by preserving the tooth's biological integrity. Furthermore, they are associated with faster recovery times and fewer complications, making them a favorable option for both the patient and the clinician. Selective endodontics, in particular, highlights the potential of tailored treatments that balance the need for intervention with the preservation of tooth vitality, supporting the notion that less invasive approaches can often provide equally, if not more, effective results.

## Author Contributions

**Sajedeh Namaei Ghasemi:** conceptualization, data curation, funding acquisition, investigation, project administration, resources, writing – review and editing. **Paria Molaei:** formal analysis, methodology, resources, software, validation, visualization, writing – original draft.

## Funding

The authors have nothing to report.

## Consent

Written informed consent was obtained from the patient to publish this report in accordance with the journal's patient consent policy. It should be noted that this form was signed by the patient's parents, as she was under 18.

## Conflicts of Interest

The authors declare no conflicts of interest.

## Data Availability

The dataset(s) supporting the conclusions of this article are available from the corresponding author upon reasonable request.
